# What Is an L-Cell and How Do We Study the Secretory Mechanisms of the L-Cell?

**DOI:** 10.3389/fendo.2021.694284

**Published:** 2021-06-08

**Authors:** Rune E. Kuhre, Carolyn F. Deacon, Jens J. Holst, Natalia Petersen

**Affiliations:** ^1^ Department of Obesity Pharmacology, Novo Nordisk, Måløv, Denmark; ^2^ Department of Biomedical Sciences, Faculty of Health and Medical Sciences, University of Copenhagen, Copenhagen, Denmark; ^3^ Novo Nordisk Center for Basic Metabolic Research, University of Copenhagen, Copenhagen, Denmark; ^4^ School of Biomedical Sciences, Ulster University, Coleraine, United Kingdom; ^5^ Department of Obesity Biology, Novo Nordisk, Måløv, Denmark

**Keywords:** L-cell, GLP-1 - glucagon-like peptide-1, experimental - animal models, *in vitro* model, hormone secretion, peptide expression

## Abstract

Synthetic glucagon-like peptide-1 (GLP-1) analogues are effective anti-obesity and anti-diabetes drugs. The beneficial actions of GLP-1 go far beyond insulin secretion and appetite, and include cardiovascular benefits and possibly also beneficial effects in neurodegenerative diseases. Considerable reserves of GLP-1 are stored in intestinal endocrine cells that potentially might be mobilized by pharmacological means to improve the body’s metabolic state. In recognition of this, the interest in understanding basic L-cell physiology and the mechanisms controlling GLP-1 secretion, has increased considerably. With a view to home in on what an L-cell is, we here present an overview of available data on L-cell development, L-cell peptide expression profiles, peptide production and secretory patterns of L-cells from different parts of the gut. We conclude that L-cells differ markedly depending on their anatomical location, and that the traditional definition of L-cells as a homogeneous population of cells that only produce GLP-1, GLP-2, glicentin and oxyntomodulin is no longer tenable. We suggest to sub-classify L-cells based on their differential peptide contents as well as their differential expression of nutrient sensors, which ultimately determine the secretory responses to different stimuli. A second purpose of this review is to describe and discuss the most frequently used experimental models for functional L-cell studies, highlighting their benefits and limitations. We conclude that no experimental model is perfect and that a comprehensive understanding must be built on results from a combination of models.

## The Physiological Function of GLP-1 and Pharmacological Utilization of GLP-1

Glucagon-like peptide-1 (GLP-1) is an intestinally produced hormone secreted by the L-cell; it is released in response to meal intake (1–3) and plays an important role in glucose homeostasis. GLP-1 lowers postprandial glucose levels by potentiating glucose-stimulated insulin secretion and by inhibiting postprandial glucose absorption rate through delaying gastric emptying (2–4). In addition, GLP-1’s glucose lowering effects are further amplified by a concurrent inhibition of glucagon secretion. This is particularly important in subjects with type 2 diabetes, as they often have inappropriately elevated levels of glucagon in spite of hyperglycemia; the hyperglucagonemia, in turn has been demonstrated to contribute importantly to the hyperglycemia (5–10).

Accordingly, the glucoregulatory actions provide the peptide with a considerable potential for the treatment of type 2 diabetes, and GLP-1 based therapies have now been part of standard care for its treatment for more than a decade (11). More recently, because of the appetite inhibiting effects of GLP-1, GLP-1 based therapies have been approved for treatment of obesity and currently represent the most efficacious pharmacological therapy for weight loss (12).

However, the native GLP-1 peptide is unsuitable as a drug, as it is rapidly degraded upon secretion by the ubiquitous proteolytic enzyme, dipeptidyl peptidase-4 (DPP-4), which cleaves intact GLP-1 (7-36amide/7-37) into the truncated metabolite, GLP-1 (9-36 amide/37) (1, 13–15). Therefore, GLP-1 receptor (GLP-1R) targeting strategies are based on the use of either DPP-4 inhibitors to prolong the half-life of the endogenous GLP-1, or on GLP-1 receptor agonists (GLP-1RAs) that are DPP-4 resistant per se and/or become DPP-4 resistant when bound to a larger protein such as albumin (1, 13, 14).

In addition to the insulinotropic and appetite-inhibiting actions, GLP-1 based therapies are also being investigated for potential effects on Alzheimer’s disease (16), nonalcoholic steatohepatitis (17), and cardiovascular risk. In particular regarding cardiovascular risk, the evidence of beneficial effects is strong, with several large outcome studies showing risk reduction for cardiovascular death, nonfatal myocardial infarction and nonfatal stroke (18–23). When combined, these clinical data therefore suggest that the therapeutic use of GLP-1 based drugs may increase in the future. Accordingly, as the source of endogenous GLP-1, the L-cell and its physiology take on renewed significance.

## What Is an L-cell?

### Morphological Definition of L-Cell

**Figure 1:**
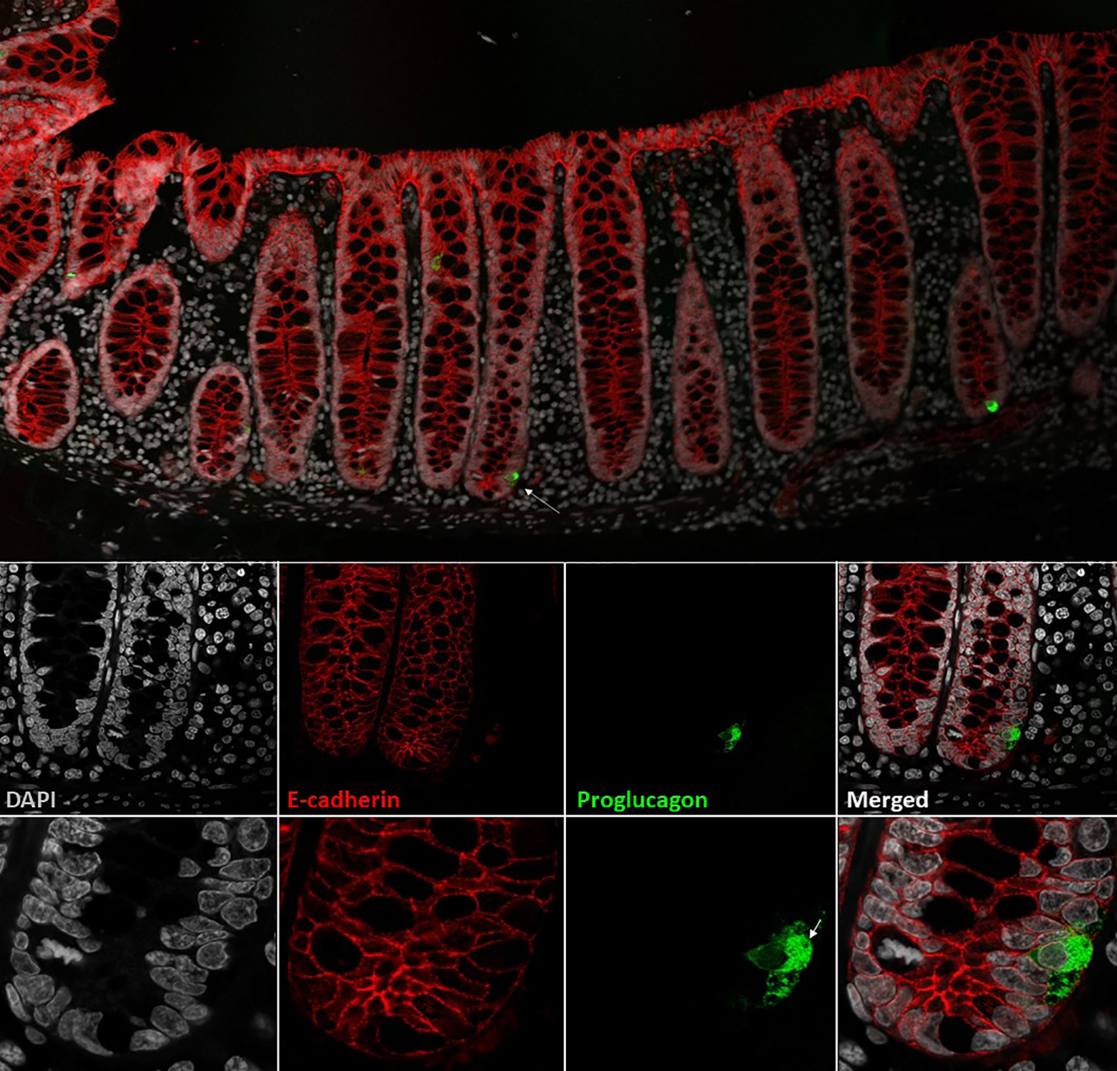
L-cells in non-human primate colon (Cynomolgus macaque). L-cells were identified based on proglucagon immunoreactivity (green). In upper panel two L-cells are shown. Lower panels shows a close-up of the L-cell in the upper panel (indicated by arrow). At the highest magnification, the individual GLP-1 granules are visible. Cell outlines are labelled by e-cadherin (red) and nuclei are stained with DAPI (grey). Cynomolgus necropsy and tissue collection was conducted at Charles River Laboratories, Montreal, Canada, according to regulations specified under the Protection of Animals Act by the Authority in the European Union (directive 2010/63/EU). Tissue samples were stained with antibodies against proglucagon (rabbit-anti-glucagon,Glu001, NovoNordisk A/S) and ecadherin (610182, BD Transduction Laboratories) detected with Cy3 and Cy5 conjugated secondary antibodies raised in donkey (Jackson ImmunoReserach) with DAPI nuclear contrast agent. The tissue section was imaged on a Leica TCS SP8 laser scanning confocal microscope with 10x/0.40 and 63x/1.30 objectives.

The classification of different enteroendocrine cells was originally based on their electron microscopy characteristics and on their hormone content by immunostaining (24, 25). Thus, cholecystokinin (CCK)-containing cells were classified as I-cells (26, 27) and GLP-1 as L-cells, whereas glucose-dependent insulinotropic peptide (GIP) was designated as being produced in K-cells and neurotensin (NT) in intestinal N-cells (28). In addition, the intestinal mucosa was noted to harbor endocrine cells that resembled the α-cells of the endocrine pancreas (29). However, later research, using electron microscopy and immunogold labeling, showed that, in contrast to α-cells, L-cells (sometimes called enteroglucagon cells) have large dense core granules which appear homogeneous, without the typical halo formation of the α-cell (30, 31). Like the majority of enteroendocrine cells, L-cells are open-type endocrine cell and have a cone-shaped appearance, with the base resting on the basal lamina of the intestinal epithelial lining. Microvilli protrude from the apical projections into the intestinal lumen, and hormone-containing granules are situated on the basolateral side facing the capillaries (32). A representative image of the L-cell morphology is provided in **Figure 1**.

### Which Peptide Are Present in L-Cells?

**Figure 2:**
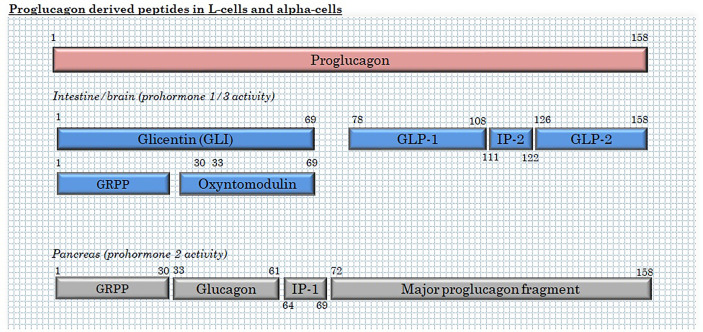
Products of proglucagon processing in L-cells and α-cells. GRPP, glicentin-related pancreatic polypeptide; and IP, intervening peptide.

L-cells are mainly classified by their production of the hormone precursor, proglucagon – a 160 amino acid pro-peptide encoded by the proglucagon gene (33), located on chromosome 37, 2q36(2). After translation, proglucagon undergoes tissue-specific processing through site-specific cleavage by prohormone convertase 1/3 (intestine and brain) or prohormone convertase 2 (pancreatic islets), cleaving at different sites to yield glicentin, GLP-1 and GLP-2 in the intestine and brain and glicentin-related pancreatic polypeptide (GRPP), glucagon and major proglucagon fragment in α-cells (34, 35) (**Figure 2**). In the intestine, further enzymatic activity cleaves part of the glicentin (about a third) into oxyntomodulin and GRPP (36, 37). In addition to these major L-cell and α-cell products, N-terminally elongated glucagon (glucagon 1-61) has been detected in human plasma (38).

In addition to the proglucagon-derived peptides, immunohistochemical studies revealed that L-cells also co-express PYY (peptide YY) in distal (ileal and colonic), but not proximal, L-cells (31, 39–41), and that a smaller sub-population of (the very few) duodenal L-cells may co-express GIP (31, 42–45). Newer research, based on cell sorting of murine cells expressing fluorescent markers under the control of the promotor coding for either CCK (46), GIP or GLP-1 (47, 48) have challenged the one hormone-one cell type dogma further. Such studies have provided evidence that enteroendocrine cells often express a broader repertoire of gut hormone genes than was appreciated earlier (49). This is particularly the case for L-cells from upper part of the small intestine, which, in the mouse, also express mRNA transcripts for CCK, GIP, NT and secretin (46, 48) in addition to proglucagon. However, the extent to which these transcripts are actually processed to mature peptides varies considerably. Thus, further immunohistochemical characterization showed that, for example, CCK was detected in all proximal L-cells, whereas GIP was only detected in only 15% of the proglucagon positive cells (48). And in spite of this, CCK and GLP-1 are not secreted in parallel from the upper gut (39). By comparison, L-cells from the distal small intestine and from the colon showed a more restricted repertoire of prohormone mRNAs, expressing mainly *Gcg*, *Cck* and *Pyy*, and with about 40 and 10% of GLP-1 positive cells also staining for PYY and GIP, respectively. Neither CCK nor GIP are secreted to any appreciable extent from the colon, however. Detailed gene profiling (48, 50) revealed that duodenal L-cells have more similarity to duodenal K-cells than they have to ileal and colonic L-cells, while the latter are more similar to each other than to the duodenal L-cells (48, 50). In comparison to the mouse, GLP-1/GIP co-localization is rare in the rat, while PYY tends to be present in more L-cells throughout the intestine, with CCK being restricted to the upper intestine (39). Further studies on isolated perfused intestinal segments from the upper or lower half of the small intestine showed that these differences in co-localization translate into differences in secretory profiles, with L-cells from the proximal small intestine secreting GLP-1, whereas L-cells from the distal half secrete both GLP-1 and PYY (39). It, therefore, appears that in both rats and mice, at least two sub-populations of L-cells exist in the small intestine. Interestingly, transcriptome analysis of fluorescence-activated cell sorted (FACS) human enteroendocrine cells recently showed that the expression patterns of mRNA precursors for gut peptides down the human intestine largely match the pattern found in mice (51).

### Distribution of the L-Cells Along the Intestine

The distribution of L-cells in the intestine has been assessed in immunohistochemical studies, showing that L-cell density (number of L-cells/mucosal area) in mice, pig and humans is low in the upper small intestine and increases down the length of the intestine to the rectum (28, 31, 42, 43, 52–54). However, such studies are laborious and, therefore, usually rely on examining sections of tissue that only cover a small fraction of the overall area and length of the intestinal mucosa. Even for small animals like the mouse, the studies usually include small specimens from selected anatomical regions (e.g. duodenum, jejunum ileum and colon), providing limited coverage of the entire intestine. Immunohistochemical analyses are, by default, two-dimensional and do not routinely take into account the difference in villus surface (the third dimension), which varies considerably down the intestine. Moreover, various methods of tissue preparation, antigen retrieval and specificity of the employed antibody may all confound interpretation of the results. As such, immunohistochemical studies are not ideal for *quantitative* assessment of L-cell density/total L-cell number down the gut. To overcome some of the limitations of immunohistochemical studies with regards to the third dimension (villus size), other studies have characterized the concentration of extractable GLP-1 in different parts of the intestine in mice, rats and pigs rather than detailing L-cell density. These studies showed that in mouse intestine, GLP-1 concentrations increased gradually from the duodenum towards the distal colon, in agreement with previous immunohistochemical studies. In rats and pigs, however, a different pattern was seen. In rats, GLP-1 concentrations peaked in the distal ileum and proximal colon, with no significant differences between jejunum, caecum and distal colon, whereas for the pig, concentrations were similar from the distal ileum to the distal colon, but below the detection limit in the duodenum and jejunum (pig) (55). However, despite having overcome the limitation with regards to the third dimension, this extraction study (55) is still limited by being based on the analysis of relatively few tissue samples covering a small fraction of the total gut mucosa, while differences in segmental length were also not taken into account. A more true and fair assessment of total L-cell distribution may be gained from two other studies. In one, L-cell numbers were directly quantified by mathematically unbiased stereology throughout the entire rat small intestine, while in the second, segmental GLP-1 content from the upper jejunum to the rectum in humans was calculated by combining published information on L-cell densities (31, 56–58) with data on respective segmental weights (59). By these approaches, the study in rats showed that L-cells were distributed rather evenly throughout jejunum, ileum and colon (52), whereas total GLP-1 concentrations in humans were found to be 3-5 times higher in jejunum and ileum compared to colon and rectum (60). Therefore, although L-cell density may be highest in colon, the significant higher surface area in the small intestine means that the upper small intestine have significant L-cell numbers despite relative low density.

### L-Cell Differentiation Pathway and Regulation of Proglucagon Production

All epithelial cells in the intestine, including the L-cells, originate from a single type of intestinal stem cell positioned in the bottom of the crypt. These Lgr5+ (leucine-rich repeat-containing G-protein coupled receptor 5)-expressing stem cells (61) are guided by paracrine factors from neighboring Paneth cells to enter the fast-dividing pool of non-specified progenitors (transit-amplifying cells). Further development of secretory cells is then initiated through inhibition of Notch signaling followed by expression of Atoh1, as reviewed elsewhere (61). Already in the crypt, some of these cells differentiate into L-cells, guided by a number of transcription factors (62). Neurogenin 3 induces endocrine specialization (63), while NeuroD1 (64), Arx and Rfx6 direct the specific L-cell development (65). Several other homeodomain proteins (such as Isl1, Cdx-1 and Pax6) have been found to promote Gcg expression by interacting with motifs of the gcg promoter elements and, theoretically, can drive the L-cell specification, but this role was confirmed *in vivo* for only a few of them (as reviewed elsewhere) (66). Apparently, effectors of the Wnt pathway (which are provided by Paneth cells in the crypt) also have positive effect on Gcg expression (67).

Preproglucagon expression increases as the cell “travels” from the bottom of the crypt up to the villus, and, as mentioned above, the cell’s hormone profile changes, possibly influenced by mediator gradients secreted by neighboring cells, which trigger the expression of other hormones. For studies of these events, an appropriate *in vitro* model and a suitable L-cell identifier such as expression of a fluorescent protein are essential to enable analysis of the transitions (65, 68, 69).

From a therapeutic point of view, these guiding cues may potentially be exploited to increase the number of functional L-cells pharmacologically in order to increase the total GLP-1 pool, and indeed, pharmacological inhibition of Notch or ROCK (Rho-associated coiled-coil-containing protein kinases 1 and 2) signaling in mice and human intestinal organoids has been shown to increase the L-cell number several fold with a corresponding increase in GLP-1 secretion (70). Application of short chain fatty acids (SCFAs), certain bile acids or GPBAR1 agonist may also increase the number of L-cells *in vitro* and *in vivo* in mice (71). Even more impressive results can be achieved *in vitro* using a combination of Notch, Wnt and MEK inhibitors (72). However, although these preclinical studies are encouraging, the safety and effectiveness of these methods for increasing the GLP-1 pool have not yet been investigated sufficiently for human trials.

In their natural environment, L-cell numbers could be regulated by feed-forward “on demand” signaling [72], presumably to cope with increasing caloric load. This is supported by reported observations of increased numbers of L-cells in individuals living with obesity and in obese rodents (73, 74). However, other studies reported reduced numbers of L-cells and their functional markers in response to short term high fat diet (up to two weeks) in mice (75) and in zebrafish (76). Consistent with the latter observation, most studies have found secretion of L-cell products to be characteristically decreased in individuals living with obesity (77, 78). Thus, the time span and capacity for L-cell adaptation requires further study. Bariatric surgery is another condition that may alter L-cells numbers. In rodents, bariatric surgery (Roux-en-Y gastric bypass) was associated with a doubling of proglucagon expression and L-cell numbers in the region of the gut that was exposed to nutrients. L-cell density was, however, not different. Rather, the increases were found to be driven by mucosal hypertrophy (79). In humans, L-cells numbers also increased after RYGB (again in the part of the intestine still exposed to nutrients), and in this case L-cell density was also increased (80). Whether the increased L-cell numbers in the parts of the intestine still in continuity represent an increase compared to preoperative numbers (before the upper part of the small intestine was excluded and underwent atrophy) warrants further investigation. Moreover, the underlying signals mediating the increases in L-cells after bariatric surgery also needs to be clarified.

Cytokines, in particular IL6, have also been reported to increase the number of L-cells and GLP-1 production (81) [although IL-6 does not appear to potentiate meal-stimulated GLP-1 secretion in humans (82)]. This may potentially be a compensatory response to provide protection against intestinal damage and stress, given that several peptides secreted by L-cells play an important role in intestinal growth and regeneration. These include GLP-1 itself (83, 84) and its sister peptide, GLP-2, which is co-secreted with GLP-1. GLP-2 has powerful intestinotrophic activity in mice (85, 86) and rats (86–88) when administered in pharmacological doses, leading to expansion of the intestinal mucosal area and increased nutrient absorption, which is seen in both healthy animals (89) and in different rodent models of intestinal injury (90–92), including rats with surgical gut resections that mimic short bowel syndrome (93). These effects of pharmacological doses of GLP-2 are also present in humans and have been exploited for therapeutic use in patients with short bowel syndrome (94). Subsequent studies showed that once daily administration of teduglutide [a DPP-4 resistant form of GLP-2, h[Gly2]-GLP-2) (87)] resulted in increased intestinal fluid reabsorption and nutrient absorption, and led to a reduction in the requirement for parenteral support (95). Teduglutide was approved for chronic treatment of patients with severe (parenteral-nutrition-dependent) short bowel syndrome in the US in 2012 (96). While both GLP-1 and GLP-1 are involved in intestinal healing and repair (83, 84), it is still debated whether these hormones are crucially important for normal intestinal growth and adaptation (97).

*L-cell hormone expression* patterns and function seem to vary, not only as a function of the anatomical location of the cells in the intestine, but are likely also to be influenced by their state of maturation. In this context, it is worth considering that the turnover time of enteroendocrine cells is short in comparison to other endocrine cell types and in mice, for example, is about 10 days (98). L-cells are no exception to this general pattern, and appear to be fully renewed within 7 days (in mice) (99), meaning that at any given time, considerable numbers of L-cells are in different stages of maturation. A number of studies have investigated, in detail, the association and causality between certain transcription factors and translation of those into the expression of specific hormones that are produced at unique times during the L-cell’s life span (63–65). Secretin was described as being present in early L-cells (100), while neurotensin and PYY appeared in mature L-cells, as indicated by the presence of these hormones higher up along the crypt-villus axis (47). As discussed above, the extent to which these different non-proglucagon hormone precursor RNAs are translated into functional peptides, however, warrants further investigation and, more importantly, the quantitative importance of L-cell derived “non-L-cell peptides” for total circulating concentrations remains to be clarified.

## Stimulation of GLP-1 Secretion as a Therapy for Obesity and Type 2 Diabetes

### The Sensory Machinery of L-Cells

GLP-1 is a classical postprandial hormone and diurnal dynamics of plasma GLP-1 concentrations thus follow meal intake patterns (101, 102). The tight coupling between meal intake and GLP-1 secretion is, at least partially, driven by direct nutrient sensing of L-cells during the phase of nutrient absorption. Over the last two decades, considerable effort has been invested into uncovering the molecular sensors involved, and important nutrient sensors (different G-protein coupled receptors and molecular transporters) expressed by the L-cells have now been described. It has long been assumed based on the morphological appearance, that the apical microvilli of the L-cells that protrude into the intestinal lumen are somehow capable of directly sensing the nutrients in the luminal content (31) to induce postprandial GLP-1 secretion. However, it was not until the development of the three GLP-1 secreting cell lines GLUTag, NCI-H716 and STC-1 in the 1990s (discussed later) (103–106) that the molecular and cellular mechanisms of nutrient-stimulated secretion began to be unraveled. A comprehensive detailing of these sensors is outside the scope of the current review, but excellent reviews can be found elsewhere (107, 108). In short, the L-cell senses nutrients *via* a number of nutrient-specific mechanisms that span from substrate uptake *via* electrogenic transporters to various G-protein coupled receptors linked to different effector proteins (Gq and Gαs).

Briefly, some nutrients, such as glucose and di-/tri-peptides, induce GLP-1 secretion through electrogenic effects and membrane depolarization (109–114), whereas lipids are thought to stimulate secretion by activation of various G-protein-coupled receptors, depending on their length. As shown *in vitro*, SCFAs stimulate GLP-1 secretion *via* activation of FFAR2 and FFAR3 (115–117) or *via* events secondary to intra-cellular metabolism (isolated perfused rat colon) (117), but only appear to do so in concert with cAMP generation by other factors. In humans, neither fermentation nor direct intracolonic application of SCFAs cause measurable increases in circulating GLP-1 (118–121), although PYY was found increased in two of the studies (120, 121). Long chain fatty acids may stimulate secretion *via* activation of FFAR1 (117, 122) although this is not easy to demonstrate in humans. Monoacyl glycerols (MAGs) however appear to stimulate secretion *via* activation of GPR119 (123), and bacterial metabolites such as indole (124), a metabolite produced from tryptophan, and S-equol (125), and prebiotics (126, 127) also appear to stimulate GLP-1 release. Furthermore, bile acids stimulate secretion of GLP-1 by activation of TGR5 receptors (128–132). However, as discussed next, the secretory responses of L-cells to these different secretagogues are not uniform, and the magnitude of the response appears to be largely related to their location in the gut.

### L-Cell Responses and Their Nutrient Exposure in Different Parts of the Intestine

Interestingly, L-cell characteristics vary along the intestine, not only with respect to co-expression of non-proglucagon derived peptides, but also with respect to the secretory responses elicited by different stimuli. Thus, duodenal and jejunal L-cells are considered generally to be more nutrient-responsive than L-cells in the colon, and they are thought to be responsible for the immediate GLP-1 response to nutrient intake that occurs within 10 min after food ingestion (101, 102). As such, the amplifying effect of GLP-1 on insulin secretion (peaking 15-30 min after food ingestion) would be expected to be mediated by GLP-1 predominantly secreted from the proximal intestine. Nevertheless, comparison of plasma GLP-1 responses to isocaloric glucose infusion into either the duodenum or the jejunum shows that the GLP-1 secretory capacity of the small intestine and its insulinotropic action increases in the distal direction (133). Importantly, GLP-1 responses to nutrients were similar from isolated perfused segments of the upper or lower small intestine of rats (whereas PYY was only secreted from the lower segment) (39). Furthermore, it was observed that GLP-1 secretion correlated tightly with the glucose absorption rate (134).

With regards to postprandial GLP-1 secretion, it is important to mention that only the L-cells in the small intestine are likely to be directly stimulated by meal intake, in spite of the many L-cells found in the colon. This is related to the fact that most macro-nutrients are absorbed in the proximal small intestine so that the colonic L-cells are not exposed to direct nutrient stimulation from the lumen. Instead colonic L-cells may be exposed to significant amounts of secondary bile acids, SCFAs and other microbial metabolites which can stimulate or modulate colonic GLP-1 secretion, albeit with a considerable delay compared to the initial intake of food. This variation in direct exposure appears to resonate into differential expression of nutrient sensors and secretory responses between L-cells in the small intestine and colonic L-cells. For instance, studies on perfused intestine preparations have shown that while glucose is a powerful stimulator of GLP-1 secretion in the small intestinal segment, colonic GLP-1 secretion is stimulated to only a small extent by glucose but is, instead, robustly stimulated by bile acids (109, 130, 134). Thus, it would be logical to propose that colonic L-cells rather function as sensors for microbiota products rather than sensors of the main macronutrients. As mentioned above it is also important to realize that any response from the colonic cells would be much delayed or unrelated in time to nutrient intake. The range and amounts of microbial metabolites reflect the diversity of the microbial community, which also shows associations with metabolic diseases and dietary intake (135) and, thus, metabolic state of the host. As the microbial community and its metabolites are unlikely to change acutely after a nutrient intake, the colonic L-cells are presented with less fluctuation in levels of stimulants, and GLP-1 release from this anatomical region is, therefore, likely to be more steady.

### Therapeutic Potential of Stimulation of Endogenous GLP-1 Secretion

The use of GLP-1 receptor agonists and the DPP-4 inhibitors have clearly demonstrated that targeting the GLP-1 axis has a great therapeutic value for T2D and obesity treatment (13, 136, 137). However, it could be speculated that a similar effect could be brought about by pharmacological stimulation of L-cell secretion (particularly if used in combination with a DPP-4 inhibitor to prevent the subsequent degradation of GLP-1). This approach could potentially be particularly effective, since stimulation of L-cell secretion will not only release GLP-1, but also, as mentioned, other anorectic peptides (neurotensin, peptide-YY, oxyntomodulin) which may be co-stored and co-released with GLP-1 (28, 46, 48).

For stimulation of endogenous L-cell secretion for therapeutic purposes it is necessary that a sufficient pool of GLP-1 is available for pharmacological targeting. Indeed, the existence of such a reserve pool is supported by both pre-clinical and clinical studies. In vitro secretion studies of primary mucosal mouse cultures have, for instance, shown that the amount of GLP-1 secreted over a 2 hour period of glucose stimulation results in a drop of only 4-10% of the total GLP-1 content (73). Similarly, in our own laboratory, we have never been able to deplete GLP-1 stores from isolated perfused rodent intestines, even after prolonged, repeated stimulation using powerful GLP-1 secretagogues [e.g. glucose and bile acids (109, 129)]. Collectively, therefore, these studies suggest that the diminished GLP-1 responses observed in individuals living with obesity (77, 78) are unlikely to be due to depleted stores. This is consistent with human studies showing that greatly enhanced GLP-1 secretion can elicited by increasing nutrient delivery rate to the small intestine by e.g. nutrient instillation *via* intestinal intubation or in conditions with an accelerated gastric emptying, such as vagotomy with pyloroplasty, gastrectomies or after bariatric surgery, which may augment responses up to 30-fold (138–145).

These findings have led to several attempts to stimulate the secretion of endogenous GLP-1 as an alternative to using exogenous administration of GLP-1 analogues. For instance, an orally available, potent, and selective partial-agonist of FFAR1 (TAK-875, or fasiglifam) reached phase three of clinical development (146) [but was terminated after concerns regarding liver safety (147)] and orally available and potent agonists of the bile acid-sensitive receptor TGR5 have also been developed (132).

## How to Best Study the Secretion of GLP-1?

Mechanistic studies of GLP-1 secretion rely on human studies, *in vivo* studies in animals and *in vitro* models (cell lines, primary epithelial cultures, isolated perfused intestine, etc.). As described in the following sections, the different models have their benefits and limitations (also summarized in **Table 1**). Therefore, to increase mechanistic depth and potentially provide human relevance application of more than one model is appropriate.

**Table 1:** Frequently used models to study L-cell secretion.

Studies on humans
*Advantages:*
- Human relevance.
- Physiologically relevant.
- Large plasma volumes can be obtained, enabling quantification of multiple hormones/molecules.
- Hormone assays are mostly readily available.
- Confounding stress-induced effects play less of a role.
- No anaesthesia required.
- Allow high temporal resolution.
*Limitations:*
- Minimal experimental control.
- Intracellular L-cell signalling cannot directly be investigated.
- Expensive.
- Time consuming (need for ethical approval and study organization).
- Inter-individual variation may be considerable, causing need for high group numbers to obtain statistical power.
- Degradation and clearance of peptides may lead to underestimation of hormone secretion.
***In vivo* animal studies**
*Advantages:*
*-* Physiologically relevant.
- Allow post-mortem studies on tissue (e.g. gene expression, protein content, histology).
- Less stringent ethical regulations than in human studies with regards to pharmacological compound use.
- More experimental control than studies in humans.
- Allow relatively quick and inexpensive genetic modification
*Limitations*
- Limited experimental control (although more control than in human studies).
- Confounding factors (e.g. stress-responses) may influence results.
- Intracellular L-cell signalling cannot directly be investigated.
- Considerable inter-animal variation requires high group numbers to obtain statistical power.
- Strain and housing conditions may profoundly affect results: results are not always reproducible between laboratories.
- Low volume plasma samples in mice limits time-resolution and number of molecules that can be quantified.
- Suitable assays may not always be available.
- Degradation and clearance of peptides may lead to underestimation of hormone secretion.
- Long term studies and studies on genetic modified animals are relatively expensive.
**Immortalized L-cell cell-lines**
*Advantages:*
- Direct L-cell sensing and secretion can be studied.
- High throughput.
- Inexpensive and easy to maintain.
- Intracellular signalling (e.g. calcium dynamics) can be studied.
- Allow for quick and inexpensive gene editing
- High concentration range for compound testing
- Large sample volume yield.
- High degree of standardization and low experiment-to-experiment variation.
*Limitations:*
- Low physiological relevance.
- Not identical to native L-cells in all aspects.
- Cells are non-polarized and lack influence from enteric nerves and paracrine signalling.
- Stimulation through physiological route (lumen or vasculature) is not possible.
- Hormone output is often insufficient to allow dynamic incubations (perifusion studies).
- Accumulation of secreted products and metabolites may influence the results.
**Primary mucosal cultures**
*Advantages:*
- Direct L-cell sensing and intracellular signalling can be studied.
- Gene editing (e.g. by siRNA) is limited.
- L-cells presumably resemble native L-cells to a larger extend than L-cell cell lines.
- Inexpensive.
- Relatively high throughput.
- Applicable for studies on human tissue.
- Applicable for studies on GMO.
- High degree of standardization and low experiment-to-experiment variation.
- High concentration range of test compounds can be applied.
*Limitations:*
- Low physiological relevance
- Duodenal and jejunal mucosa is challenging to maintain in culture.
- Cells are non-polarized and without influence from enteric nerves and paracrine signalling.
- Stimulation through physiological route (lumen or vasculature) is not possible.
- Hormone output is often insufficient to allow dynamic incubations (perifusion studies).
- Accumulation of secreted products and metabolites may influence the results.
- Experiments are done on fragile mucosal preparations susceptible to apoptosis.
**Gut tissue specimens**
*Advantages:*
- Studies are done on fresh tissue: Less changes in L-cell physiology.
- L-cells maintain their polarization and are integrated into the epithelial lining.
- High sample volume.
- Applicable for studies on human tissue.
- Applicable for studies on genetic modified animals.
- L-cells resemble native L-cells to a larger extend than L-cell cell lines.
- High concentration range of test compounds can be applied.
*Limitations:*
- Low physiological relevance.
- Specimens have a short survival time and ensuring adequate oxygen supply to crypt cells may be a challenge.
- Hormone output is often insufficient to allow dynamic incubations (perifusion studies).
- Accumulation of secreted products and metabolites may influence the results.
- Stimulation through physiological route (lumen or vasculature) is not possible.
- The extent to which enteric nervous signalling is maintained is uncertain.
**Organoids**
*Advantages:*
- Allow gene editing.
- Real time L-cell monitoring.
- Investigation of intracellular L-cell signalling.
- Maintain cell renewal, epithelial lining integrity and paracrine signalling.
- Allows for studies on polarized monolayers and 3D structure.
- High concentration range of test compounds can be applied.
*Limitations:*
- Do not fully mimic the intestinal environment, resident cells and blood vessels.
- Do not form complete villus compartment.
- L-cell responsiveness may be affected by cell culture conditions.
**Ussing chambers**
*Advantages:*
*-* Studies are done on fresh tissue and native L-cells.
- L-cells maintain their polarization and are connected to the same cells as they were *in vivo*.
- Applicable for studies on human tissue.
- Tissue can be stimulated from the physiological relevant route (apical side or basolateral side).
- Applicable for studies on genetic modified animals.
- High concentration range of test compounds can be applied.
*Limitations:*
- Tissue do not survive well in chambers: relatively short time window for doing experiments.
- Human specimens may be difficult to obtain.
- The extent to which enteric nervous signalling is maintained is uncertain.
- Hormone output is often insufficient to allow dynamic incubations or perifusion studies.
- Accumulated secretion products may influence the results.
- Gene editing is not possible.
- Not suitable for investigation of intracellular L-cell signalling.
**Isolated perfused intestines**
*Advantages:*
- High degree of physiological relevance and anticipated translation to *in vivo*.
*-* Studies are done on fresh tissue: no significant changes in L-cells.
- L-cells maintain their polarization and are connected to the same cells as they were vivo.
- Allow for stimulation *via* the physiological relevant route (lumen or vasculature).
- Allow for constant perfusion at a physiological flow rate.
- Secretion can be studied at a high time resolution (down to second intervals).
- Absorption of nutrients can be directly be investigated.
- Large sample volume yield.
- Enteric nerve signalling and peristaltic movements are largely preserved.
- Applicable for studies on genetic modified animals.
- High concentration range of test compounds can be applied.
*Limitations:*
- Requires a certain level of surgical skills.
- Relatively expensive.
- Laborious and not applicable for screening purposes.
- siRNA mediated knock down of target genes is not readily possible.
- Relatively short time window for doing experiments (usually up to four hours).
- Not suitable for investigation of intracellular L-cell signalling.

### Studies in Humans

Studies in humans are the only way to provide definitive experimental answers on human physiology. Indeed, the experiments that confirmed the existence of the incretin effect were based on studies carried out in humans (148, 149). Since then, studies in humans have contributed indisputably to the general understanding of L-cell physiology, and have been instrumental in deciphering the stimuli that causes GLP-1 secretion in humans (2, 3), and the extent to which each macronutrient evokes GLP-1 secretion (139). Studies involving procedures more invasive than the simple ingestion of a certain nutrient(s) can also be performed in humans. For instance, infusion of the GLP-1R antagonist Exendin-9 has been used to investigate the relative importance of GLP-1 secretion on postprandial gastric emptying rate, - glucose absorption and -glucose excursions (150), and the role of exaggerated GLP-1 secretion for improved β-cell function and glucose tolerance after Roux-en-Y gastric bypass (151). They have also shown that the incretin effect is reduced in humans living with type-2-diabetes (152), and that this is due to loss of GIP’s (153, 154), but not GLP-1’s, insulinotropic activity (155–157). This is a significant discovery that shaped the direction of further incretin research. However, as important human studies are for generating data on human physiology, they have obvious limitations with respect to the level of control (invasiveness) that can ethically be justified and detailed mechanistic aspects of L-cell physiology and L-cell secretion therefore has to rely on other experimental models. Intracellular L-cell signaling can, for instance, not be investigated *in vivo* and direct stimulation of the vascular side of the gut (through the mesenteric artery) is not ethically justifiable. Naturally occurring mutations can, to some extent, be useful for studying the effect of a certain receptor, sensor, enzyme, etc. on L-cell secretion, and have, for instance been used to elucidate molecular mechanisms involved in the appetite and weight reducing effects of GLP-1 based treatment in a certain patient population (158). However, only a few individuals carry mutations in the target of interest, and results from such experiments are more difficult to interpret, as the magnitude of any change of function resulting from naturally occurring mutations varies. Studies carried out in humans have obvious advantages over rodent studies, in that the risk of any species differences confounding interpretation of results is eliminated, and that relatively large plasma samples can be obtained, allowing several hormones/molecules to quantified in the same sample. However, human studies are expensive and time-consuming, and usually run over several years.

### *In Vivo* Animal Studies

Animal models, particularly mice and rats, are commonly used for studying aspects of L-cell physiology, where clinical studies have limitations. One major benefit of using mice as study animals is that there are endless opportunities for genetic modification (including single nucleotide editing), allowing assessment of the impact of specific target genes on GLP-1 secretion. Countless gene knockout and knock-in studies have revealed the molecular mechanisms underpinning GLP-1 secretion induced by bile acids, glucose, SCFA and proteins, as thoroughly reviewed elsewhere (107). However, a clear limitation of these studies is the small volume of blood which can be withdrawn from a mouse during a GLP-1 secretion test, which restricts temporal resolution. Furthermore, quantification of L-cell peptides in plasma is not trivial, as not all assays, including the GLP-1 assays, are reliable with respect to accuracy, sensitivity and specificity (159, 160). In the case of GLP-1, secretion is best estimated by measuring “total GLP-1” i.e. the sum of intact GLP-1 (of which there is usually very little) and its primary metabolite, GLP-1 9-36amide (or metabolites, since GLP-1 9-36amide may also be broken down to small fragments) (160). This requires assays that can measure both the intact peptide and the metabolite(s), and is possible with assays directed against the amidated C-terminus (161). GLP-1 also exists as a glycine-extended isoform (GLP-1 7-37) but this does not circulate in appreciable concentrations in humans (57). Murine and human GLP-1 are identical (in fact all mammalian GLP-1s are similar) (34, 162) and the mouse GLP-1 is also predominantly amidated (163), but in contrast to humans, the primary metabolite (9-36amide) is processed further, within minutes, by endoproteolytic cleavage by NEP 24.11 (164). Sandwich assays directed at GLP-1 9-36amide will, therefore, vastly underestimate GLP-1 secretion. Thus a GLP-1 response to an OGTT in mice is detectable with 9-36amide sandwich assays for only 6 minutes after glucose administration, whereas the response is much bigger and lasts for at least 30 minutes when measured with a C-terminally directed GLP-1 assay (which also detects the fragments from NEP-mediated degradation) (164). Currently, no low-volume C-terminal assays are available and measurement of GLP-1 secretion in mice, therefore, relies on just a few, or perhaps just a single terminal sample, which makes assessment of the time-course of the GLP-1 responses to a given stimulus difficult. Because of their larger size, rats (typically 10-15 times larger than mice) do not generate the same problems. However, unlike mice and humans (57, 163), rats do not amidate GLP-1 as effectively, with about 35% being glycine-extended. This has implications for the choice of assay (163, 165), but probably does not have physiological consequences, as glycine-extended GLP-1 and amidated GLP-1, at least in humans, have similar elimination half-lives and equal effects on the endocrine pancreas and on cephalic phase acid secretion (i.e. regulation of parasympathetic nervous activity) (166, 167). A more general limitation of *in vivo* studies is the level of experimental control that can be achieved, albeit that experiments in e.g. anaesthetized animals will allow more invasive approaches. For instance, administration of a test compound *via* a physiologically relevant route (e.g. the vascular supply of the gut) cannot always be easily done, while intra-cellular signaling pathways cannot directly be investigated. Additionally, pharmacological tools, such as blockers of molecular sites that may be part of the secretory pathway, cannot always be used *in vivo* since they may be toxic in the intact animal. For example, we used KCl, lidocaine (a blocker of voltage-dependent sodium channels, veratridine (activator of voltage-dependent sodium channels), and 2-4-dinitrophenol (mitochondrial uncoupler that, in high concentrations, blocks mitochondrial ATP-generation) in the isolated perfused rat small intestine model (109) to explore the mechanisms underlying glucose-stimulated GLP-1 secretion, but the concentrations required to be effective would, most likely, be lethal in an *in vivo* model. In anesthetized animals, the anesthetic itself may also confound the study by influencing neuronal regulation and/or directly or indirectly, affecting physiological processes. For instance, anesthetics can increase blood glucose and influence gastric emptying, intestinal motility and insulin secretion, and the effects may vary according to the feeding status and the type of anesthetic used (168–176). The confounding effects of anesthetics on the secretion of GLP-1 have not been studied in detail, but are likely to be strong and similar to the changes in blood glucose excursions after an OGTT, which are dramatically influenced by most frequently used types of anesthetics: hypnorm/midazolam, ketamin/xylazin, pentobarbital or isoflurane), presumably as a result of pronounced interference with neuronal regulation of secretion and gastrointestinal motility (176). Finally, variation in housing and experimental conditions and animal-to-animal and strain-to-strain variation are significant general limitations of studies in rodents, causing many studies to involve too few animals to allow for definitive conclusions, and reproducibility between laboratories to be a challenge (177). Even when housing conditions and experimental procedures are standardized, the choice of species and strains may still profoundly affect results. For example glucose tolerance significantly varies between four commonly used inbred mouse strains (178), despite the use of standardized housing and experimental conditions.

## *In vitro* Models to Study GLP-1 Secretion and L-Cell Characteristics

### Immortalized L-Cell Cell-Lines

As mentioned above, different GLP-1 secreting cell lines originating from different species have been developed. Studies using these cell lines have been instrumental in demonstrating that L-cells can directly sense glucose and secrete GLP-1 in response, and they provided the first evidence that L-cells are electrically excitable (179). In addition, the GLP-1 secreting cell lines also allowed additional studies of the molecular sensors responsible for direct L-cell stimulation by other types of nutrients, as thoroughly reviewed elsewhere (107). However, although GLUTag and STC-1 cells exhibit many similarities to native L-cells, they also diverge in a number of aspects. Thus, altered expression of some G-protein-coupled receptors (48) and hormone content (180). The GLUTag cell actually in many ways resemble I-cells more than L-cells, and the STC-1 cells resemble K-cells [e.g. they contain more GIP than GLP-1 (180)]. Therefore, data from the cell lines must be interpreted with caution, but also provide an inexpensive and high throughput platform for screening purposes. Further analysis may then be done, using other experimental models (discussed below) with greater physiological relevance.

### Primary Mucosal Cultures

During the last 15 years, primary intestinal epithelial cultures have been widely used for studies on GLP-1 secretion and intra-cellular L-cell signaling. In particular, epithelial primary colonic mouse cultures, e.g. (44, 47, 71, 114, 115, 181), and primary colonic human cultures (182, 183), which are more robust in culture than small intestine epithelium (184), have been used and have partially replaced the use of GLP-1 secreting cell lines. Small intestinal epithelial cells release proteolytic enzymes which may complicate the experimental conditions and cell survival. In addition, small intestine cultures begin to undergo apoptosis once the villus structure is disrupted. The use of primary cultures has, therefore, mainly been restricted to studies of sensing and signaling in the more resilient colonic L-cells. However, as mentioned earlier, colonic L-cells (and distal small intestinal L-cells) differ from the proximal L-cells with regard to co-expression of non-proglucagon-derived hormones as well as expression of nutrient sensors. Accordingly, therefore, cultured colonic L-cells may not be the optimal model for studying meal-related GLP-1 secretagogues. For instance, the GLP-1 response elicited by glucose is considerably smaller in this model, both compared to that from isolated perfused mouse and rat small intestines (109, 134, 185), as well as that observed in humans (after intake of a standardized glucose containing solution, 75g) (139). An advantage of cultured colonic L-cells, however, is that they, like the GLP-1 producing cell lines (44), permit intracellular signaling and electrophysiological signaling to be studied directly (44, 51). However, they only provide a small window for cell differentiation studies, as the cells lose their ability to proliferate, making these cell lines unsuitable for studies on L-cell development and differentiation.

### Organoids

**Figure 3:**
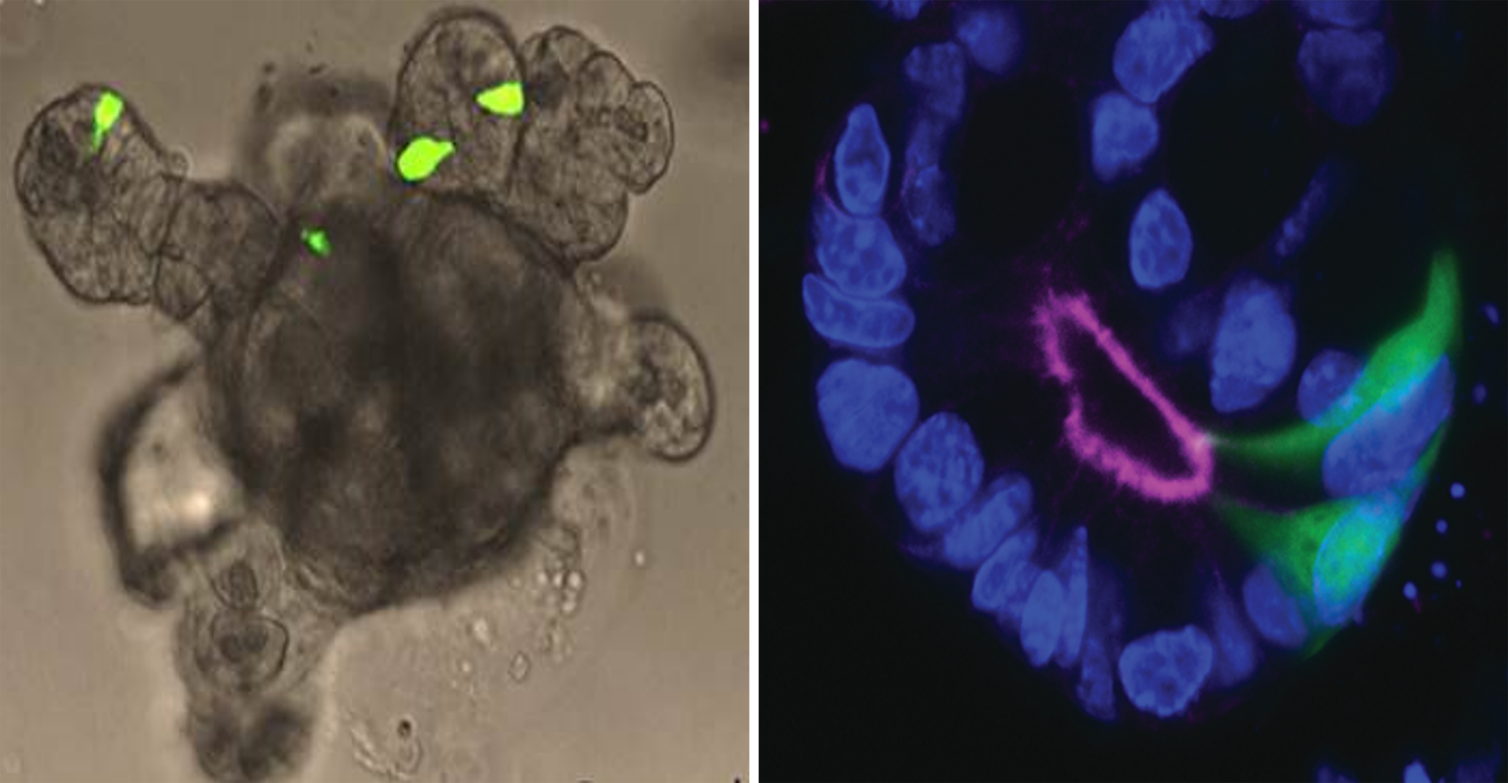
Intestinal organoid. Left panel: Small intestine organoid generated from Glu-Venus mouse. L-cells are labelled by expression of Venus (shown in green) and have cone-like appearance similar to native L-cells in intact mucosa. Right panel: L-cells (green) in an organoid crypt. Luminal side and apical surface of cells is outlined by F-actin staining. Cell nuclei are labeled by DAPI (blue).

Organoids are self-organized cell aggregates, which can be generated from primary cultures containing stem cells or from induced or embryonic stem cells to resemble miniature organs or tissues. Accordingly, they are a popular platform for studies on tissue development. However, because organoid cells differentiate from stem cells *in vitro*, and the precise factors guiding differentiation and maturation of cells are often unknown, the functional capacity of the intact tissue is poorly mimicked in some organoids systems. Fortunately, human (186) and mouse intestinal organoids display a high degree of functionality and can be generated using just a small number of growth factors (187). These self-organizing structures accurately reconstruct the small intestinal epithelial layer, and have similar rates of renewal and cell composition, including L-cells. Traditionally, the organoids can be grown as three-dimensional structures embedded in an extracellular matrix (187), but can also be grown as monolayers (188), or as gut-on-chip containing permeable membrane and controlled *in vivo*-like microenvironment by perfusion and providing intestinal peristalsis-like motions and flow (189). Organoid studies have provided new knowledge on the mechanisms of intestinal cell differentiation and renewal (61). As other cell types, functional L-cells are generated in organoids (63) and small intestine organoids are now used in studies on secretion of GLP-1 and other intestinal hormones (70, 188, 190, 191). If the organoids are prepared from cells from GLP-1 reported mice with fluorescent L-cells it is possible to easily identify the L-cells in the organoid (see **Figure 3**). Now, with the use of precision gene editing at specific genomic loci, such as CRISPR, which uses RNA-guided endonucleases, such as Cas9, real-time L-cell studies have become possible also in primary human organoid cultures generated from patients. Thus, a study on CRISPR-Cas9 engineered primary human ileal organoid cultures showed that L-cells could be identified and characterized with respect to gene expression and intracellular signaling (electric signals and intracellular calcium levels), allowing simultaneously investigation of intracellular signaling and L-cell secretion (188). Another advantage of organoids is that these self-renewing cultures can be maintained for years, as was shown for mouse organoids (192). When it comes to human cell-based organoids, neither primary nor human induced-pluripotent stem cell-derived organoids (193) effectively mimic the self-renewing fully functional gut epithelium, but provide valuable insight into developmental enteroendocrine cell biology aspects (65, 193). Compared to primary mucosal cultures (which will be discussed next), organoids are capable of forming a ‘mini-lumen’, thereby providing a polarized cell layer (i.e., cells with a luminal and a basolateral side) (61, 194) (as illustrated in **Figure 3**). However, because of its small size, it can be difficult to access the luminal side of organoids in order to stimulate receptors and transporters that are expressed on apical side of the cells (195). Recently, substantial progress has been made to alleviate this limitation, by generating scaffold-based intestinal cultures. This technique relies on using a silicone (or similar material) matrix, where the stem cells can be seeded; these then develop into crypt-like invaginations of the matrix and proliferating cells form a monolayer of intestinal epithelial cells (195). Such matrix-based culture systems would not only allow for intra-luminal stimulation, but would also permit non-intestinal epithelial components (such as fibroblasts, immune and endothelial cells) to be introduced into the tissue, which may help bring the functionality closer to the native state.

In addition to studies on GLP-1 secretion, organoids also allow for tests on paracrine GLP-1 and GLP-2 signaling in the intestine, due to colocalization of the different cell types in the organoid. Moreover, organoids in several ways have an advantage over ex-vivo and primary cultures, because they lend themselves to plasmid, virus or RNA-mediated gene manipulation, thanks to the possibility of long term maintenance in culture with preserved functionality.

### Gut Tissue Specimens (Intestinal Fragments)

GLP-1 secretion has also been studied in human gut tissue biopsies or surgical specimens taken from duodenum, ileum and colon (111, 196). As for the other *in vitro* platforms, test compounds can readily be applied to dissected intestinal fragments to elicit GLP-1 secretion. The major benefit of the gut tissue specimen model is that it allows for direct studies of human L-cells without prior cultivation. As such, these studies are less laborious, and since the tissue is processed immediately after isolation and the behavior of the L-cell in the fragments is presumably similar to L-cells in situ. Tissue handling and the integrity and health of the studied tissue, however, needs to be monitored closely to assure that data derived using this methodology are meaningful. It is also misleading to study luminal stimuli with these fragments, which will expose mainly the basolateral aspect of the cells and are not protected by the mucus and glycocalyx etc. like the small luminal processes of the endocrine cells.

### Ussing Chambers

Ussing chambers, designed by the zoologist Hans Ussing in 1950, are a classical model for studying gastrointestinal physiology and pharmacology such as ion secretion (measured indirectly by transepithelial resistance), paracellular flow, and to some extent intestinal permeability (e.g. passage of fluorescently labeled bacteria) (197). However, Ussing chambers have also been used to study hormone mechanisms of L-cell secretion (128, 198–202). Technically, Ussing chambers involve mounting mucosal tissue segments in specialized chambers, enabling live quantification of the trans-epithelial resistance and potential difference of the mounted specimen. While these parameters may not be strictly relevant for L-cell secretion, they provide an indication of integrity of the mounted tissue. As with the perfused intestine model (described next), the epithelial barrier in this model is maintained, enabling administration of test compounds specifically to either the luminal or basolateral side of the epithelial layer. However, it comes at the cost of a relatively short window of time for experiments, as the mounted tissue usually only remains viable for a few hours. This is particularly the case for upper small intestinal tissue, which seems less resilient than tissue from more distal regions (203). Studies on L-cell secretion by Ussing chambers are, therefore, most often performed on ileal/colonic tissue specimens and investigations are often restricted to include just a few test compounds at a time. Unlike isolated perfused intestines, but as with studies on cell lines, primary mucosal cultures and organoids, incubations are typically performed under static conditions for up to a few hours, which bears little resemblance to normal physiology, where peristaltic movements constantly move luminal chyme down the intestine and where blood perfusion rapidly removes secreted molecules (e.g. GLP-1) as well as waste products. Moreover, this model, like the organoid model, lacks extrinsic factors which influence intestinal function, e.g. autonomic nerves.

### Isolated Perfused Intestines

**Figure 4:**
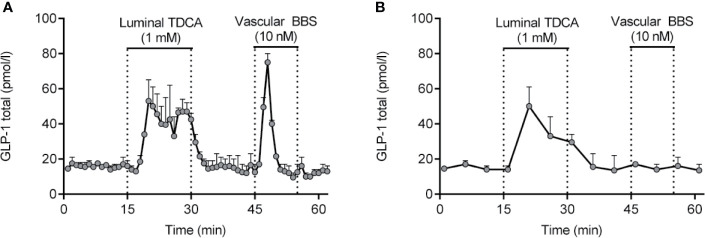
GLP-1 secretion from isolated perfused rat small intestine (lower half) in response to luminal infusion of taurodeoxycholic acid (TDCA) and vascular (inter-arterial) infusion of bombesin (BBS). Samples were collected at minute intervals, allowing the short lasting, but pronounced, GLP-1 response to BBS to be identified **(A)**. Had a sampling frequency of 5-min intervals instead been used **(B)**, the GLP-1 response would not have been noticed. Methods used are described in details elsewhere (204). Data are presented as means+SEM, n = 2. are presented as means+SEM, n = 2.

Isolated perfused intestines from different species have been used for decades for studying basic gut physiology, including nutrient absorption and gut secretion (ions and hormones). Experimentally, isolated perfused intestines can be prepared in a variety of ways, but the core concept is the same; namely to isolate and perfuse the organ by cannulation of the supplying arteries and collection of the venous effluent. As such, this approach is particularly suitable for investigation of dynamic events, such as absorption (e.g. glucose) or gut secretion (e.g. GLP-1) (110, 134, 204). Because of the minimal manipulation of the gut itself, the strength of this experimental model is that the vasculature and cytoarchitecture are preserved, whereby this model becomes particularly physiologically relevant (204). This means that all relevant interactions, both paracrine, neuronal and interactions *via* for instance gap junctions are preserved in the model. As an example of this, blockade of somatostatin signaling by infusing a somatostatin-receptor antagonist increases GLP-1 secretion from the isolated perfused mouse small intestine to a large extent (205). Moreover, because the anatomical relationships are undisturbed in the isolated perfused intestine, this model allows for administration of test compounds *via* the physiological relevant route, enhancing the translatability of the results. For instance, we have used the isolated perfused intestine to demonstrate that bile acids stimulate secretion of GLP-1 by activation of basolateral sensors (TGR5) (128, 129), SCFAs stimulate secretion *via* basolateral FFAR1 (GPR40) (206), glucose exclusively stimulates secretion *via* a luminal pathway and requires SGLT1 mediated uptake (109) while peptone-mediated secretion depends on absorption and activation of basolateral calcium-sensing receptors (110). The most important aspect of this model is the preservation of secretory dynamics, both in terms of magnitude and timing, due to the natural perfusion of the tissue. This not only ensures adequate respiration and removal of waste products, but also ensures adequate export/removal of secreted products, as opposed to the accumulation which occurs in interstitial tissues in static incubations. Note that *perifusion* of tissue specimens only solves this problem with regards the most superficial cell layers; for the majority of the tissue, conditions remain static. Another benefit of isolated perfusion models is the time resolution that this model offers, enabling secretory dynamics to be studied in detail according to how frequently effluent (venous) samples are collected (we normally collect samples at minute intervals). This time resolution ensures that short lasting, but potentially pronounced, secretory responses are not missed, which could occur under conditions of static incubations where short lasting responses may be ‘lost’ in the background basal secretion during the incubation period (normally 1-2h) (see example in **Figures 4A, B**).

From an analytical point of view, the isolated perfused intestine model(s) also offers benefits compared to *in vivo* rodent studies, since large sample volumes (determined by perfusion flow) may be collected (204, 205) and because GLP-1 concentrations are higher than in peripheral systemic plasma, because of sample collection before dilution in the splanchnic and systemic circulation. Nevertheless, the isolated perfused intestine model does also have limitations. The surgical procedure requires some practice, and experiments are laborious compared to *in vitro* studies; both during the actual experiment and regarding the subsequent sample analyses (since a substantial number of samples usually are generated). The isolated perfused intestine is, therefore, not the ideal experimental tool for screening purposes or for other experimental designs requiring high throughput. Moreover, intra-cellular signaling (for instance calcium dynamics) cannot be directly investigated, and siRNA mediated knock-down is not possible (but knockout animals are readily studied). Inhibition of targets (receptors, transporters, ion channels, etc.) therefore has to rely on either infusing inhibitors or on genetic knock out. With these limitations in mind, the isolated perfused intestine model may be considered a useful model to bridge and expand upon observations made *in vitro* and *in vivo*.

## Concluding Remarks

The original definition of an L-cell was based on electron microscopical appearance and immunohistochemical stainings, characterizing L-cells as open-type enteroendocrine cells with large dense core granules and containing GLP-1 and/or the other proglucagon-derived peptides (glicentin, oxyntomodulin and GLP-2). It turned out that GIP may also be found in a in small subset of proximal L-cells, while PYY is found in most of the distal L-cells. During the last decade, detailed expression analyses of sorted primary L-cells have shown that L-cells are not as uniform as previously thought. Instead, they vary, depending on their anatomical site as well as their position along the crypt/villus axis, with respect to expression of prohormone transcripts and hormone content, as well as to their expression of nutrient sensitive G-protein-coupled receptors. L-cell expression of molecular sensors responsible for macronutrient-stimulated GLP-1 secretion also varies depending on the macro-anatomical location resulting, for example, in a loss of glucose responsiveness in colonic L-cells, but strong responses to microbiota metabolites, such as secondary bile acids. These previously underappreciated features of L-cell functionality make it clear that the original characterization of L-cells as a uniform cell type needs to be revised, and that the L-cell population needs to be subclassified. In this regard, we suggest to sub-classify L-cells into at least two different populations based on their differences in expression profiles and functional characteristics – proximal and distal L-cells. Moreover, such sub-classification(s) should also take into account that proximal and distal L-cells presumably serve different functions in terms of metabolic regulation. Thus proximal L-cells appear to be the main sensors and responders to nutrient ingestion, and are, as such, responsible for most of the postprandial rise in GLP-1 secretion, whereas distal L-cells are likely to function as metabolic sensors that register overall basal levels (for example, bacterial metabolites). This distinction is important for both biological studies as well as the search for new pharmacological targets. With evolving understanding of L-cell(s) transcriptomics and functionality, biological and pharmacological studies could benefit from even further sub-classification.

Given the substantial stores of endogenous GLP-1, pharmacological mobilization of these reserves holds a potential promise as an alternative to current GLP-1R based treatment strategies for type 2 diabetes and obesity, and may potentially prove to be more efficacious than GLP-1R mono-therapy, since it would be accompanied by co-secretion of other anorectic hormone products of the L-cell. Harnessing endogenous GLP-1 for therapeutic purposes, however, requires detailed knowledge of the molecular sensors responsible for GLP-1 secretion and, because of the differences outlined above, it would be important to take into consideration which L-cell subtype is intended to be targeted (proximal or distal L-cells?, L-cells in the crypt or in the villus?). While studies in humans and *in vivo* animal models are essential for setting the direction (e.g. which molecules stimulates L-cell secretion) they usually provide limited information about the molecular mechanisms that mediate the secretory responses. However, genetically modified rodents, with deletions e.g. of a receptor of putative importance for L-cell sensing and secretion, have enabled the molecular sensing machinery of the L-cell to be studied to some extent *in vivo*. Detailed studies on the sensing machinery of the L-cell, L-cells differentiation and the factors involved, however, requires use of other models.

While immortalized GLP-1 secreting cell lines paved the way for studies on L-cell sensing machinery, and remain an important tool for high-throughput (screening), development of more advanced and more physiologically relevant experimental models provided further understanding of L-cell sensing and function. These include in particular (1) primary mucosal cell cultures from mouse and human colon, (2) mouse and human intestinal organoids, (3) human intestinal gut specimens, and (4) isolated perfused mouse and rat small intestines. In addition to GLP-1 secretion studies, cell based models (1 and 2) allow for intracellular cell signaling studies and gene editing. Tissue-based models (3 and 4) are more restricted in terms of investigation of intra-cellular signaling, but benefit from having maintained the natural L-cell polarization (2,3 and 4) and cell contacts with no or minimal alterations induced by culturing conditions. In the isolated perfused intestine model, test compounds can be administered at the site of the gut where they would normally occur in highest concentration (e.g. luminal glucose), rendering this model the one that it is closest to normal physiology, while also offering a high temporal resolution (by the minute). The drawback with this model is that it is not suitable for screening purposes or for direct investigations on intra-cellular events. In summary, each of these experimental models has its own benefits and limitations. Accordingly, the best and most thorough study approach is to combine the different experimental models to generate the most detailed and physiologically representative data.

## Author Contributions

REK concepted idea. REK and NP drafted the first version of the manuscript. CD and JH provided important intellectual content to the first draft and subsequent versions. All authors contributed to the article and approved the submitted version.

## Funding

RK was supported by postdoctoral scholarships from the Lundbeck Foundation (R264-2017-3492) and from “Købmand i Odense Johann og Hanne Weimann, f. Seedorffs Foundation”. Furthermore, this work was supported by another grant to REK from Lundbeck Foundation (R289-2018-1026).

## Conflict of Interest

RK and NP are employed by Novo Nordisk. Novo Nordisk manage had no influence on the conception or content of this review.

The remaining authors declare that the research was conducted in the absence of any commercial or financial relationships that could be construed as a potential conflict of interest.
